# Chitosan Oligosaccharides Modulate Macrophage Inflammatory Signaling: Molecular Docking and Transcriptomic Evidence

**DOI:** 10.3390/ijms27177775

**Published:** 2026-08-30

**Authors:** Yujun Sung, Siriporn Namwongsa, Sineenart Songkoomkrong, Supawadee Duangprom, Napamanee Kornthong

**Affiliations:** 1 Research Unit in Innovative Marine Biotechnology and Natural Bio-Resources for Sustainable Health and Wellness, Thammasat University, Pathum Thani 12120, Thailand; ethansung416@gmail.com (Y.S.); siriphorn.nonkhaow@gmail.com (S.N.); sineenartsong@gmail.com (S.S.); su.duangprom@gmail.com (S.D.); 2 Department of Biochemistry and Genetic Engineering, Faculty of Medicine, Kasetsart University, Bangkok 10900, Thailand; 3 Chulabhorn International College of Medicine, Thammasat University, Rangsit Campus, Pathum Thani 12120, Thailand

**Keywords:** chitosan oligosaccharides, inflammation, molecular docking, oxidative stress, transcriptomics

## Abstract

Oxidative stress-induced macrophage activation and vascular injury are major contributors to atherosclerosis, a chronic inflammatory disease associated with approximately 20 million deaths worldwide. This study investigated the antioxidant and anti-inflammatory activities of structurally characterized low-molecular-weight chitosan oligosaccharides (COS) derived from mud crab (*Scylla olivacea*) shell waste by hydrochloric acid hydrolysis and explored their molecular interactions with inflammation-related targets. Structural characterization by ^13^C-NMR and MALDI-TOF confirmed the identity of COS, while DPPH and ABTS analysis demonstrated concentration-dependent antioxidant activity. In LPS-induced RAW 264.7 macrophages, COS showed no cytotoxicity and significantly reduced nitric oxide production at 80 and 160 µg/mL. Molecular docking predicted favorable interactions of COS with several inflammation-associated proteins, with iNOS and COX-2 exhibiting the most favorable docking scores and extensive hydrogen-bonding and polar interactions. Transcriptomic profiling further revealed broad transcriptional remodeling and enrichment of pathways related to inflammation and atherosclerosis, including TNF, NF-κB, MAPK, Toll-like receptor, and lipid-and-atherosclerosis signaling. COS markedly suppressed inflammatory mediators, particularly *Nos2* (iNOS) and *Ptgs2* (COX-2) together with multiple cytokines and chemokines, consistent with reduced nitric oxide production and modulation of macrophage activation and foam cell-associated processes. Integration of docking and transcriptomic analyses identified iNOS and COX-2 as convergent candidate anti-inflammatory targets of COS, supporting its ability to attenuate inflammatory signaling through relevant pathways. These findings suggest that COS may modulate macrophage inflammatory responses through coordinated regulation of oxidative stress and inflammation-related signaling pathways.

## 1. Introduction

The process of atherosclerosis is an ongoing, lipid-based inflammatory process of the arterial walls which leads to approximately 20 million cardiovascular-related deaths annually throughout the world [1]. The focus on the role of inflammation in atherosclerosis was initiated with the recognition that it is not simply a passive process of lipid deposition into arterial walls but rather a chronic inflammatory condition caused by endothelial dysfunction, oxidative stress, and prolonged macrophage activity [2,3,4].

Hyperlipidemia and reactive oxygen species stimulate the endothelial surface, causing further penetration and oxidation of the low-density lipoproteins (LDL), creating modified forms of LDL (ox-LDL), which promotes further attraction of monocytes and their subsequent transformation into foam cells through the action of scavenger receptors; this is characteristic of early lesions within the arterial wall [5,6,7,8]. Due to the fact that oxidative and inflammatory signals from macrophages contribute to continued growth and expansion of the lesions, macrophages have become the primary target for the development of novel therapeutic interventions aimed at preventing or treating atherosclerotic disease.

Chitosan oligosaccharides (COS), low-molecular-weight β-(1→4)-linked D-glucosamine derivatives of chitosan, have attracted considerable attention as marine-derived bioactive compounds because of their antioxidant, anti-inflammatory, and immunomodulatory properties, as well as their ability to attenuate atherosclerosis-related pathways involved in lesion development in experimental models [9,10,11]. However, the reported biological activities of COS vary considerably among studies because many preparations differ in molecular weight, degree of acetylation, and oligomer composition, while their structural characteristics are often incompletely defined. These physicochemical differences substantially influence the biological activity of COS, making it difficult to establish clear structure–activity relationships [9,12,13].

In our previous study, we developed a simple and reproducible hydrochloric acid hydrolysis method to produce a structurally characterized low-molecular-weight COS fraction. This preparation demonstrated improved physicochemical properties that are expected to enhance cellular uptake and bioavailability. Moreover, in 3T3-L1 adipocytes, COS reduced intracellular lipid accumulation and triglyceride content while promoting lipolysis and insulin-stimulated glucose uptake, suggesting beneficial effects on metabolic dysfunction [14]. Despite these promising findings, the molecular mechanisms by which structurally defined COS regulate macrophage inflammation remain poorly understood. Most previous studies have focused on reductions in nitric oxide and pro-inflammatory cytokines as biological endpoints without identifying the molecular targets responsible for these effects or examining global transcriptional responses associated with macrophage activation, foam cell formation, and atherosclerosis [15,16,17]. Therefore, integrating transcriptomic profiling with molecular docking provides a comprehensive approach for identifying inflammation-related signaling pathways and candidate target proteins that may contribute to the anti-inflammatory activity of COS and its potential relevance to inflammation-associated diseases.

Herein we describe a method to prepare a structurally defined COS with a low average molecular weight by treatment of *Scylla olivacea* shell waste with HCl. We used NMR spectroscopy and mass spectrometry to confirm that our preparation was indeed a low-average molecular weight chitosan oligosaccharide (COS) [14,18,19]. Our COS preparation was then assessed using an integrated approach that included determining its antioxidant capabilities, assessing cytotoxicity and its ability to suppress NO in LPS stimulated RAW 264.7 cells, performing molecular docking against eleven receptors associated with both inflammation and atherosclerosis and comparing them against the known NSAID diclofenac, and conducting genome wide RNAseq analysis to assess global gene expression with subsequent enrichment analysis of the results into relevant pathways. Our goal was to integrate the predictions made by molecular docking regarding where COS would bind into macrophage cells with the observed effects of those bindings on gene expression to demonstrate that COS is capable of modulating multiple aspects of macrophage inflammation versus being a singular pathway inhibitor and therefore could be considered as a candidate for preventing atherosclerosis.

## 2. Results

### 2.1. Structural Characterization of HCL-Derived COS

The physicochemical characteristics of COS used in this study were previously reported by [14]. Briefly, MALDI-TOF mass spectrometry demonstrated that COS consisted predominantly of low-molecular-weight oligomers (approximately 300–2000 Da) with degrees of polymerization ranging from 2 to 9 (Figure S1). Structural confirmation by ^13^C NMR spectroscopy indicated that the COS was mainly composed of glucosamine units with a high degree of deacetylation (92.35%) and minor residual N-acetylglucosamine residues (Figure S2). Detailed characterization data, including MALDI-TOF MS spectra and NMR analysis, are described (Figure S1).

### 2.2. Antioxidant Activity of COS in the DPPH and ABTS Assay

The antioxidant activity of COS was initially evaluated using the DPPH radical-scavenging assay. COS exhibited an IC_50_ value of 2.86 mg/mL, indicating measurable in vitro free-radical-scavenging activity. To further characterize its antioxidant properties, the ABTS radical-scavenging assay was performed, yielding an IC_50_ value of 2.49 mg/mL. The lower IC_50_ value observed in the ABTS assay compared with the DPPH assay suggests that COS exhibited greater scavenging activity toward ABTS radicals under the conditions tested. Thus, these findings demonstrate that COS possesses antioxidant activity in vitro, as evidenced by its ability to scavenge both DPPH and ABTS radicals.

### 2.3. Effect of COS on Cell Viability of RAW 264.7 Cells

**Figure 1:**
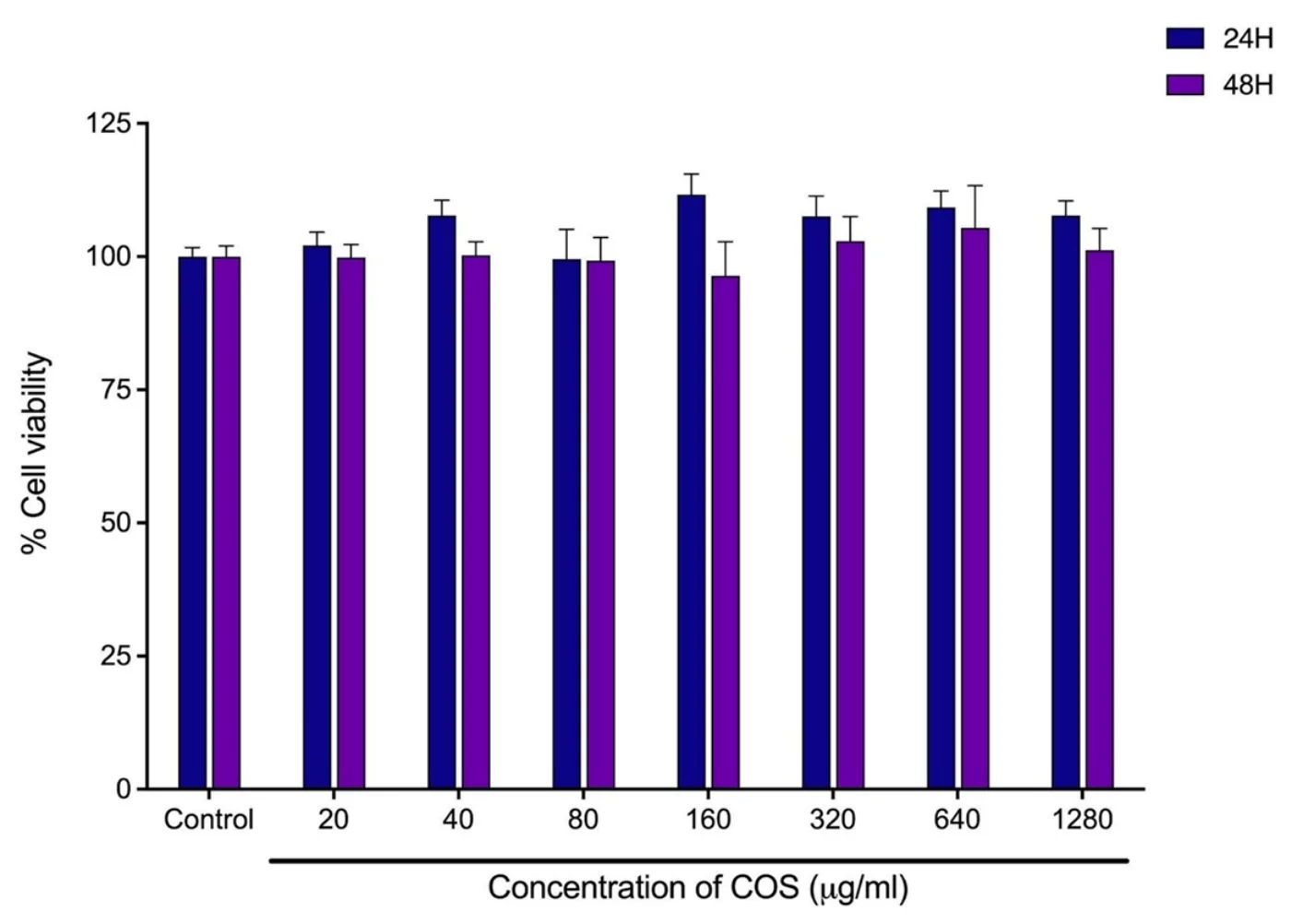
Effect of COS on RAW 264.7 cell viability. Cells were treated with COS (20–1280 µg/mL) for 24 h and 48 h; viability was assessed by MTT assay and expressed as a percentage of the un-treated control. Results are presented as mean ± SEM from four independent experiments (*n* = 4). Statistical analysis was performed using one-way ANOVA with Tukey’s multiple-comparison test.

The cytotoxicity of HCl-derived COS isoform on RAW 264.7 macrophages was assessed via an MTT assay following 24 h and 48 h treatments across concentrations between 20 and 1280 µg/mL. The result demonstrates that COS displayed no observable cytotoxicity at any concentration tested, with cell viability consistently ranging from 95% to 110% compared to the untreated control. This demonstrates that COS is biocompatible and well-tolerated by RAW cells, even at elevated doses. Furthermore, COS treatment did not induce abnormal cell proliferation, as viability values remained within normal physiological variations and did not exceed control levels in a dose-dependent manner. These results demonstrate that COS is non-toxic in RAW 264.7 macrophages, supporting its suitability for downstream anti-inflammatory assays without confounding cytotoxic effects (Figure 1).

### 2.4. Inhibition Assay of Cellular Nitric Oxide Production

**Figure 2:**
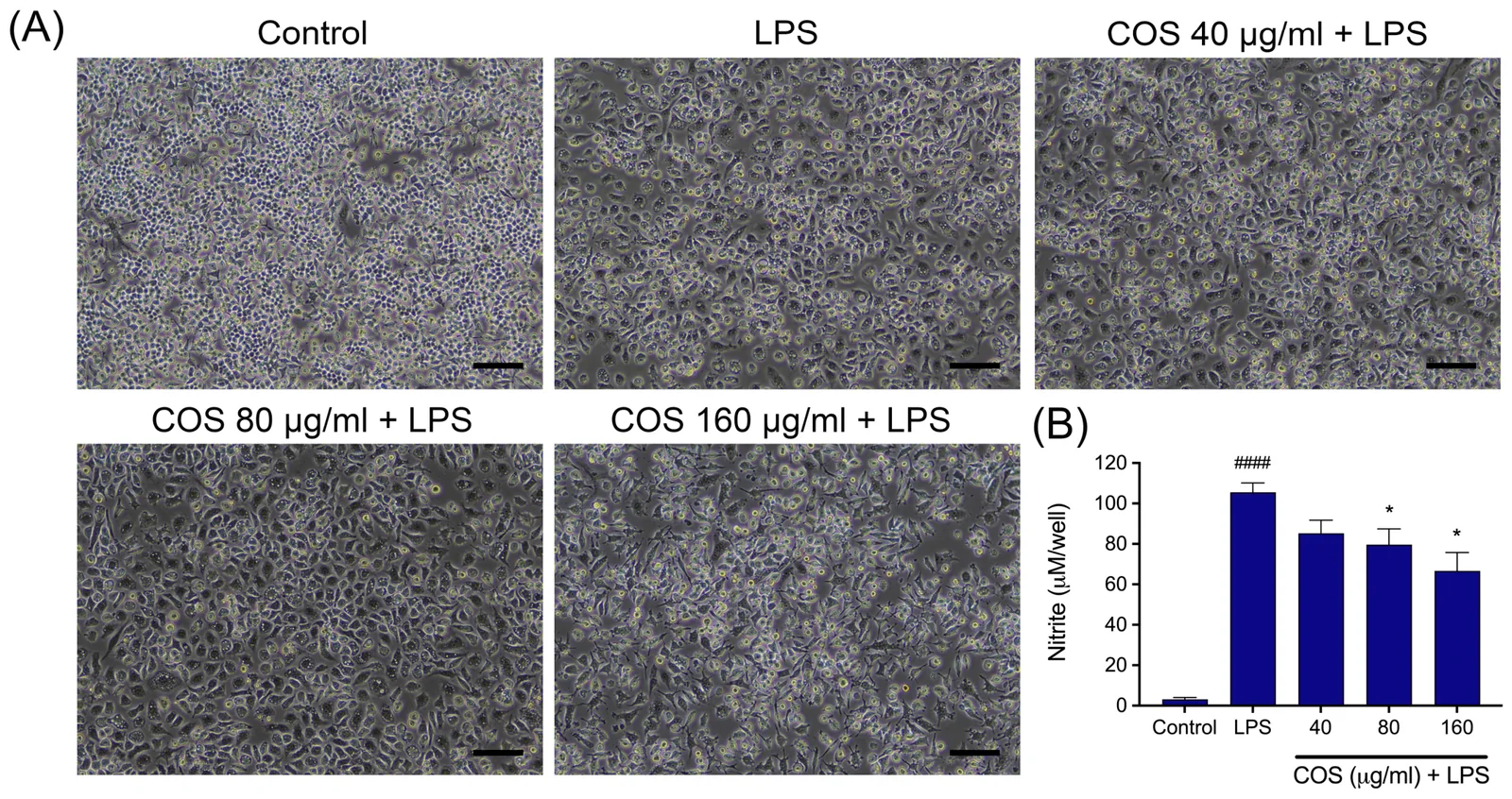
Morphology and nitrite levels of RAW 264.7 cells after pretreatment with COS. (**A**) Representative images showing the morphology of RAW 264.7 cells in the untreated control, LPS-stimulated, and COS-pretreated groups (40–160 µg/mL). Images were captured at 10× magnification; scale bars = 100 µm. (**B**) Nitrite levels in the culture medium of RAW 264.7 cells stimulated with LPS (1 µg/mL) following pretreatment with COS at concentrations ranging from 0 to 160 µg/mL. Data are presented as mean ± SEM from four independent experiments (n = 4). #### *p* < 0.0001, LPS-stimulated cells compared with the untreated control; * *p* < 0.05, COS-pretreated groups compared with the LPS-stimulated group.

The ability of COS to inhibit LPS-induced inflammatory activation was assessed by quantifying nitrite accumulation in RAW 264.7 macrophages via the Griess assay. Stimulation with LPS (1 µg/mL) markedly elevated NO production compared with the untreated control, confirming successful induction of the inflammatory response. COS treatment attenuated this LPS-induced in a dose-dependent manner. While 40 µg/mL COS produced a modest reduction in NO levels, significant inhibition was observed at 80 and 160 µg/mL, where nitrite production decreased to approximately 80 µM and 67 µM of the LPS group, respectively. These findings indicate that COS pretreatment reduced LPS-induced nitrite production in RAW 264.7 macrophages, indicating attenuation of NO production under the experimental conditions (Figure 2A,B).

### 2.5. Structural Analysis of Macrophage-Relevant Inflammatory Receptors and Molecular Docking of COS and Reference Ligands

Atomic coordinates and mature amino-acid sequences for inflammation-associated receptors related in macrophage activation and foam cell formation, including Cyclooxygenase-2 (COX-2), Fibroblast Growth Factor 1 (FGF1), Galectin-3, Heat Shock Protein 90 Alpha Family Class A Member 1 (HSP90AA1), Heparanase (HPSE), Nitric Oxide Synthase (NOS), and Tumor Necrosis Factor Alpha (TNF-α), were retrieved from the RCSB Protein Data Bank and used as receptor targets for docking of chitosan-oligosaccharide (COS). The proteins collectively integrate redox signaling (NOS), pro-inflammatory eicosanoid and cytokine axes (COX-2, TNF-α), growth-factor/trophic signal (FGF1/2), and pattern-recognition lectin signaling (galectin-3) to induce the atherogenic macrophage program.

Molecular docking was performed to investigate the potential interactions of COS with selected inflammation-associated proteins. To assess the appropriateness of the docking protocol and predicted binding cavities, experimentally characterized reference ligands present in the corresponding crystal structures were used as validation ligands [20]. Each reference ligand was docked into the same predicted binding cavity used for COS docking to assess the consistency of the docking procedure and the appropriateness of the selected binding site relative to experimentally characterized ligand-binding regions (Table 1).

Following validation of the binding sites, COS was docked independently against each receptor. COS exhibited predicted docking scores of −4.8, −6.8, −6.0, −7.7, −6.4, −7.1, and −4.8 kcal/mol for FGF1, HPSE, HSP90AA1, COX-2, TNF-α, iNOS, and galectin-3, respectively (Table 1). The predicted cavity sizes ranged from 122 to 4425, with the largest cavity observed for COX-2 and the smallest for galectin-3. Among the investigated targets, COS produced the most negative predicted docking score for COX-2 (−7.7 kcal/mol), followed by iNOS (−7.1 kcal/mol), HPSE (−6.8 kcal/mol), and TNF-α (−6.4 kcal/mol). These findings identify several inflammation-associated proteins as candidate COS-interacting targets.

### 2.6. Effects of COS Treatment on the Transcriptome of RAW 264.7 Cells

#### 2.6.1. Differential Expressed Genes and KEGG Pathway Analysis

To investigate the molecular mechanisms underlying the protective effects of COS against inflammatory stimulation, transcriptomic profiling was performed in COS-pretreated RAW macrophages followed by LPS induction compared with LPS-treated control cells.

**Figure 3:**
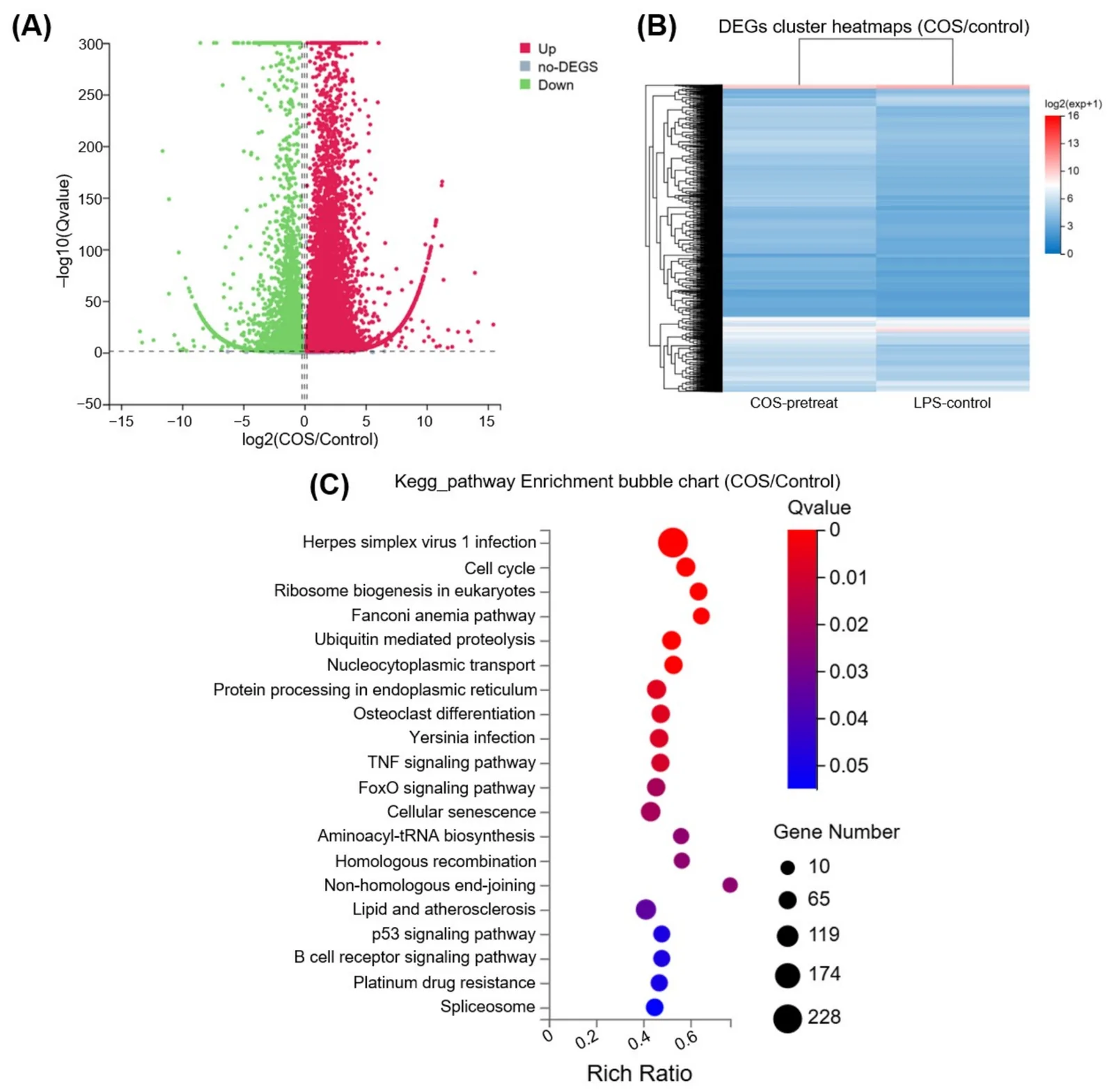
Transcriptomic analysis and KEGG enrichment of COS pretreatment in LPS-induced RAW 264.7 cells. (**A**) Volcano plot of differentially expressed genes (DEGs) between COS pretreatment and LPS-treated control. (**B**) Hierarchical clustering heatmap showing distinct transcript expression patterns between groups. (**C**) KEGG pathway enrichment analysis of DEGs showing top 20 significant enrichment of pathways.

The volcano plot demonstrated extensive transcriptomic alterations after COS pretreatment (Figure 3A). A total of 15,590 differentially expressed genes (DEGs) were identified (Table S1), including 11,399 upregulated transcripts (73.1%) and 4191 downregulated transcripts (26.9%), indicating that COS induced widespread transcriptional remodeling in macrophages under inflammatory conditions. The predominance of upregulated transcripts suggests that COS may activate multiple protective and adaptive cellular responses rather than exclusively suppressing gene expression. Hierarchical clustering analysis of DEGs further demonstrated clear separation between the COS pretreatment and LPS-treated groups (Figure 3B), indicating distinct transcriptomic signatures and confirming that COS substantially altered the LPS-induced cellular response. To understand the biological relevance of these transcriptomic changes, KEGG enrichment analysis was performed using significantly regulated transcripts (Figure 3C) (Table S2). Several pathways closely related to inflammation, macrophage activation, foam cell formation, and lipid-handing were significantly enriched. Additionally, Enriched GO terms of all significantly regulated transcripts were classified into biological processes (BP), cellular components (CC), and molecular functions (MF) (Tables S3–S5).

Among the enriched pathways, the TNF signaling pathway and NF-κB signaling pathway were particularly notable because these pathways are major regulators of macrophage inflammatory responses and are activated during LPS stimulation. Their enrichment suggests that COS pretreatment modulates inflammatory signaling networks involved in cytokine production and immune activation. Importantly, enrichment of the lipid and atherosclerosis pathway indicates that COS may regulate processes involved in macrophage lipid handling and vascular inflammation. Since excessive lipid uptake and sustained inflammatory activation promote the conversion of macrophages into foam cells, modulation of this pathway suggests a potential role of COS in reducing mechanisms associated with foam cell formation and subsequent atherosclerotic progression. Additional enrichment of pathways, including cellular senescence, protein processing in the endoplasmic reticulum, ubiquitin-mediated proteolysis, and cell cycle regulation, suggests that COS may further support cellular adaptation and restoration of macrophage homeostasis under inflammatory stress.

#### 2.6.2. Transcriptomic Identification of Key Differentially Expressed Genes Involved in Inflammatory Regulation

Transcriptomic profiling was performed to identify COS-responsive genes involved in macrophage inflammation and lipid-handing processes. The analysis focused on inflammatory regulation, foam cell formation, and lipid and atherosclerosis-associated signaling.

##### Inflammatory Relevance

Transcriptomic profiling of COS-treated macrophages revealed coordinated differential expression across the principal innate-immune and lipid and atherosclerosis-relevant pathways, including Toll-like receptor, NOD-like receptor, NF-κB, MAPK, TNF, HIF-1, and lipid-and-atherosclerosis signaling (Table 2). The strongest change was observed for *Nos2* (iNOS; log_2_FC = −7.92, Q = 1.05 × 10^−26^), consistent with the reduced nitrite production observed experimentally. The prostaglandin-synthesizing enzyme *Ptgs2* (COX-2) was also significantly reduced (log_2_FC = −0.84; FC = 0.56, Q = 5.00 × 10^−22^), corresponding to an approximately 44% decrease in transcript abundance. Other strongly downregulated inflammatory genes included the pro-inflammatory cytokines *Il1b* (−2.94), *Il18* (−1.94), and *Il6* (−1.80), the chemokine *Ccl5* (−5.42), and the stress-activated kinases *Mapk14* (p38α; −5.74) and *Mapk3* (ERK1; −1.59), all at stringent FDR thresholds. In contrast, the upstream pattern-recognition and signal-transduction apparatus was transcriptionally induced, including the Toll-like receptors *Tlr2*, *Tlr4*, and *Tlr6*, the inflammasome sensor *Nlrp3*, the adaptors *Irak4* and *Traf6*, the IκB kinase subunits *Ikbkb* (IKKβ) and *Ikbkg* (NEMO) and the NF-κB subunit *Rela* (p65), with reciprocal downregulation of the inhibitor *Nfkbia* (IκBα). The antioxidant regulator *Nfe2l2* (NRF2; +1.59) was upregulated, whereas inducible NOS (iNOS) was downregulated, possibly suppressing pro-inflammatory signaling through NF-κB, which directly regulates the expression and activity of iNOS. Taken together, COS treatment was associated with attenuation of macrophage inflammatory effector responses, consistent with the observed reduction in NO production. Although changes were observed in genes associated with the upstream TLR–NF-κB–inflammasome pathways, these findings do not confirm the inhibition of these signaling pathways. The observed downregulation of *Nos2* provides additional molecular evidence for involvement of the iNOS/NO axis in the response to COS; however, further studies are required to determine whether COS directly affects iNOS expression, enzymatic activity, or upstream signaling events.

##### Foam-Cell Formation

Within the lipid-and-atherosclerosis pathway, COS treatment produced coordinated differential expression of the nuclear-receptor and calcium-dependent transcriptional regulators that govern macrophage lipid handling (Table 3). The PPARγ–RXRα heterodimer partners *Rxra* (RXRα; log_2_FC +9.29, Q = 9.67 × 10^−58^) and *Pparg* (PPARγ; +5.24) were among the most strongly upregulated transcripts, together with the PPARγ target gene *Cd36* (CD36/FAT; +2.21), a scavenger receptor for oxidized and modified lipoproteins. The calcineurin–NFAT arm was coordinately induced, comprising the calcineurin catalytic subunit *Ppp3cb* (calcineurin Aβ; +3.97) and all three NFAT isoforms *Nfatc1* (+6.00), *Nfatc2* (+7.94), and *Nfatc3* (+2.47), indicative of activated Ca^2+^/calcineurin-dependent transcription, all at stringent FDR thresholds. In parallel, genes controlling lipoprotein uptake and reverse cholesterol transport were downregulated, including the lectin-like oxidized-LDL receptor *Olr1* (LOX-1; −4.92, Q = 1.05 × 10^−4^), the low-density lipoprotein receptor *Ldlr* (−1.41), and the efflux transporters *Abca1* (ABCA1; −0.89) and *Abcg1* (ABCG1; −1.11). The pronounced suppression of Olr1/LOX-1, the largest-magnitude change among the lipid-uptake receptors, is consistent with attenuated scavenging of oxidized LDL, a primary driver of foam cell initiation, while reduced *Ldlr* further limits receptor-mediated lipoprotein internalization. Taken together, these data indicate that COS activates the principal PPARγ–RXRα-CD36 and calcineurin–NFAT programs while inhibiting the ABCA1/ABCG1 efflux mechanism that regulates macrophage cholesterol metabolism, identifies lipoprotein uptake and cholesterol efflux. This pattern suggests, as a hypothesis for further investigation, that COS may modulate macrophage lipid-handling pathways, including cholesterol-efflux and foam cell formation assays, which is required before any effect on these pathways.

##### Lipid- and Atherosclerosis-Associated Pathways

Within the lipid-and-atherosclerosis pathway, COS treatment produced coordinated differential expression of Rho-family GTPase and actin-cytoskeletal signaling, which governs macrophage adhesion, polarization, and directional migration (Table 4). Upregulated transcripts were concentrated in the RhoA–ROCK2 contractility axis, comprising *Rhoa* (log_2_FC +3.42, Q = 2.31 × 10^−28^) and *Rock2* (+2.24), the small GTPase *Cdc42* (+1.81), and their upstream guanine-nucleotide exchange factors *Vav2* (+2.33) and *Vav3* (+2.31), indicative of enhanced activation of the GEF–GTPase circuitry that drives cytoskeletal remodeling and cell motility. Concurrent induction of the non-receptor tyrosine kinase *Src* (+1.02), the class IA PI3K catalytic subunit *Pik3ca* (+2.66), the Notch-ligand E3 ubiquitin ligase *Mib2* (+7.68), and the transcription factor *Pou2f1* (+6.89) is consistent with broad activation of receptor-proximal kinase signaling coupled to cytoskeletal output, all at stringent FDR thresholds. In contrast, the downregulated set comprised effectors of the inflammatory and extracellular-matrix-remodeling program, including the immune-restricted PI3Kδ and *Akt2* nodes *Pik3cd* (−7.33) and *Akt2* (−9.73), focal-adhesion kinase *Ptk2* (FAK; −6.42), the RhoGEF *Arhgef1* (−2.58), the type I interferon master regulator *Irf7* (−4.07), the monocyte chemoattractant *Ccl12* (−3.77), the costimulatory receptor *Cd40* (−3.59), and the matrix metalloproteinase *Mmp3* (stromelysin-1; −4.33). Downregulation of *Ccl12*, *Cd40*, *Mmp3*, and *Irf7* is consistent with attenuated chemotactic recruitment, costimulatory signaling, and ECM degradation, processes that promote monocyte infiltration and foam cell formation. Taken together, these data indicate that COS may modulate Rho-GTPase–associated cytoskeletal and inflammatory signaling in macrophages, rather than broadly suppressing these pathways.

### 2.7. Integration of Molecular Docking and Transcriptomic Profiling Identifies the Potential Anti-Inflammatory Targets of COS

To identify potential molecular targets underlying the anti-inflammatory activity of COS, docking results were integrated with transcriptomic profiling of COS-treated LPS-induced RAW 264.7 cells. Among the predicted receptors, COS exhibited the strongest binding affinity toward COX-2 (−7.7 kcal/mol), while showing moderate interaction with iNOS (−7.1 kcal/mol). Transcriptomic analysis supported these observations by revealing significant downregulation of *Nos2* (iNOS; log_2_FC = −7.92) and *Ptgs2* (COX-2; log_2_FC = −0.84). Reduced *Nos2* expression was consistent with decreased nitrite production measured by the Griess assay, whereas lower *Ptgs2* expression suggested attenuation of prostaglandin-associated inflammatory signaling. Thus, the overlap between docking targets and differentially expressed genes identified iNOS and COX-2 as candidate anti-inflammatory targets of COS, supporting subsequent analysis of their molecular binding interactions.

**Figure 4:**
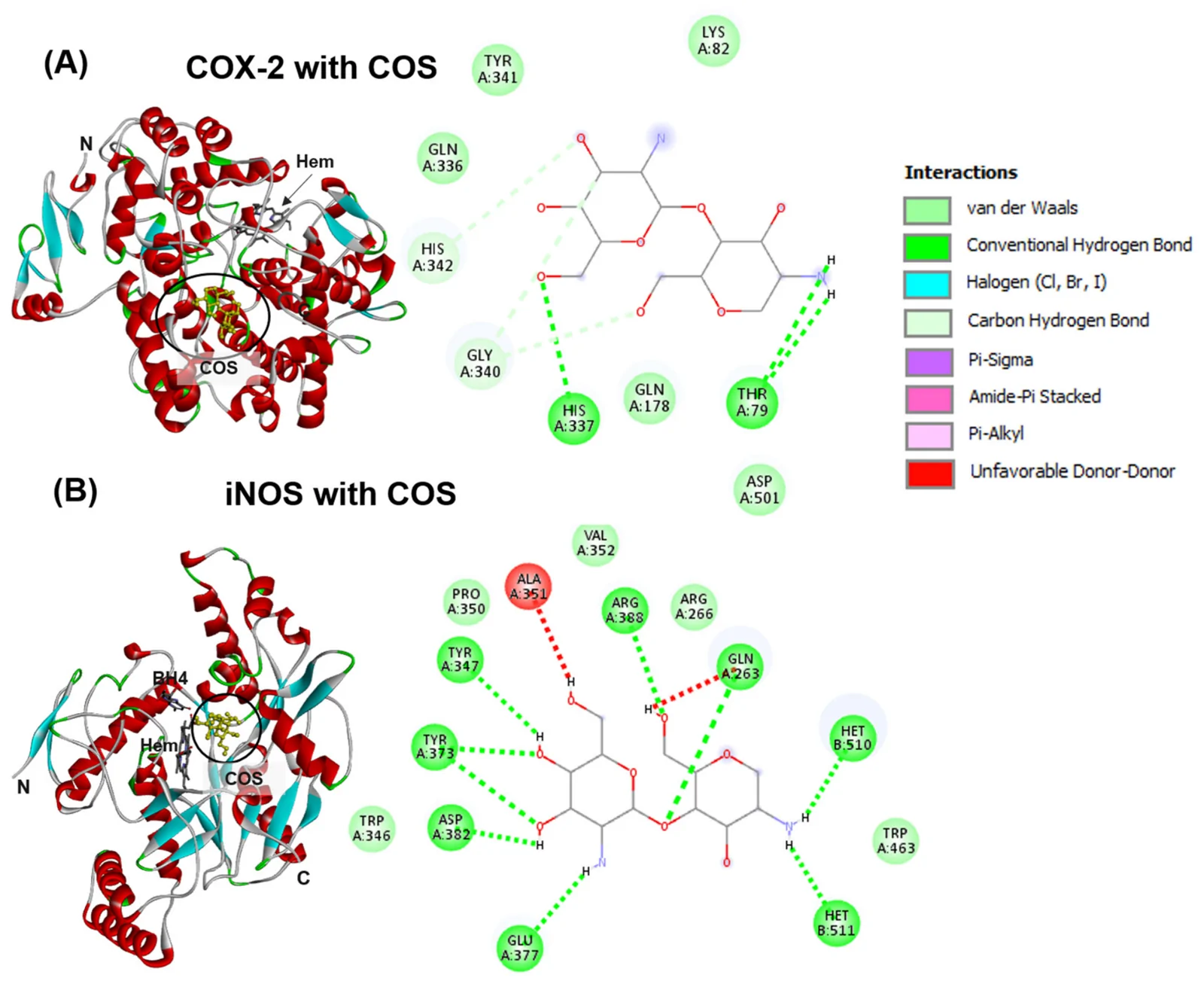
Molecular docking complexes COS with inflammation-associated receptors, including (**A**) COX-2 and (**B**) iNOS, together with their corresponding two-dimensional interaction maps. Yellow stick represents COS.

The docking complexes of COS with COX-2 and iNOS are presented together with their corresponding two-dimensional interaction maps. COX-2 showed a notable strength in forming a broader and more diversified interaction network, characterized by multiple conventional hydrogen bonds and van der Waals interactions with residues distributed throughout the pocket (Figure 4A). COS also interacted with Gln178 and Tyr341, which are key residues involved in inhibitor recognition and binding within the COX-2 active site [21]. This pattern suggests that COS may stabilize receptor binding through extensive polar contacts and flexible accommodation within the active site rather than relying predominantly on hydrophobic interactions.

Similarly, COS maintained stable binding through multiple hydrogen-bond interactions and contacts with residues located within the catalytic region of iNOS target, including Glu377, Tyr373, Tyr347, Asp382, Arg388, and Gln263, which have been reported to contribute to inhibitor recognition and selective iNOS binding (Figure 4B) [22]. The binding profile of COS indicates its potential to engage inflammatory targets through a distinct interaction mode supported by extensive polar contacts, which may contribute to stable receptor association and modulation of inflammatory signaling.

Integration with transcriptomic analysis further revealed significant downregulation of *Nos2* (iNOS) and *Ptgs2* (COX-2) following COS treatment, identifying these proteins as potential convergent targets associated with its anti-inflammatory activity. Together, the docking and transcriptomic findings suggest that COS may modulate inflammatory signaling and macrophage responses through interactions with COX-2 and iNOS.

## 3. Discussion

This study investigated the precisely defined low-molecular-weight distribution of the hydrolyzed COS prepared by hydrochloric acid that possessed antioxidant and anti-inflammatory properties. This COS obtained from mud crab shell waste could sustainably promote local and industrial economy as the daily waste was turned into a high-value biomedical material. The current study differs from many previous studies that used large, diverse and poorly characterized COS mixes. Advantages of HCl-derived COS offer a rapid, cost-effective, and highly reproducible approach compared to enzymatic digestion, oxidative degradation or chemical depolymerization [18,19]. Subsequently, the structural identity and integrity of HCl-derived COS confirmed through ^13^C NMR spectroscopy and MALDI-TOF mass spectrometry ensured reproducibility for downstream biological and mechanistic investigations [23,24]. Previous studies from Guo et al. (2018) reported that 1H NMR spectra of two COS samples, finding major signals at ~2.0 ppm (CH3 of N-acetyl group) and ~3.1 ppm (H-2 of GlcN) and calculated degrees of deacetylation (DD) of 88.4% and 87.4% respectively [9]. In this study, ^13^C NMR and MALDI-TOF were used to verify glycosidic integrity and linkage uniformity that COS used in this study contained molecular sizes ranging between 318 and 498 Da, with a 344 Da as a principal constituent identified to be D-glucosamine dimer (DP2). This guided us to predict the chain length, charge, and hydrogen-bonding networks of COS molecules obtained from this study. Since low-molecular-weight oligosaccharides exhibit better solubility in aqueous environments, enhanced membrane permeability, and superior cellular uptake in comparison to their high-molecular-weight counterparts [25,26], the predominance of dimeric DP in this study was therefore significant to be expected for more effective intracellular bioactivity. In contrast to larger polymeric chitosan which is typically restricted to extracellular or membrane-localized effects [27,28], the precisely defined low-molecular-weight COS might be readily transported through cell membranes and entered intracellular signaling pathways [29,30]. Therefore, selectively modulate excessive inflammatory signaling without inducing nonspecific macrophage suppression.

This study differs from previous reports relying on structurally heterogeneous COS mixtures or chitosan derivatives with high molecular weight where variability in the degree of polymerization and acetylation complicates mechanistic interpretation. Specifically, earlier studies demonstrated the impact of COS in suppressing LPS-induced nitric oxide, iNOS, and COX-2 expression in RAW 264.7 macrophages [16,17], as well as in reducing atherosclerotic lesion burden in ApoE^−^/^−^ mice [10] without precise structural definition. In contrast, the HCl-derived COS used in this study exhibits a narrowly defined low-molecular-weight profile with dimeric D-glucosamine, allowing accurate correlation between molecular structure and anti-inflammatory bioactivity. Low-molecular-weight COS has been reported to exhibit greater solubility and intestinal absorption than higher-molecular-weight chitosan, with negligible cytotoxicity toward Caco-2 cells [9,11,31]. These findings support the potential bioavailability of COS; however, its intracellular localization and access to membrane-associated or intracellular targets remain unclear. Therefore, the structurally characterized COS used in this study provides a relevant framework for investigating its potential effects on macrophage-mediated inflammation, while acknowledging that its cellular uptake and target engagement require further experimental validation. Further studies are therefore needed to determine whether the COS used in this study is absorbed in a similar manner.

The COS prepared in this study was proven for its antioxidant and anti-inflammatory properties. Since the basic mechanism of anti-inflammatory action of COS remains poorly defined [3,32] and existing works rarely identify the molecular receptors through which COS exerts these effects and few studies have contextualized COS activity within macrophage-driven atherogenesis [12,16]. In this study, we speculated that COS played its intracellular actions by infiltrating into macrophage cells due to its small-size molecular properties. This was also reported in studies of COS in cellular uptake, where COS effectively entered the cells take action [29,30,33]. COS displayed non-cytotoxic and non-proliferating effects, confirming high biocompatibility of its molecules, and this property is confirmed by other COS studies [28,33]. Oxidative stress and macrophage-mediated inflammation are important processes associated with vascular inflammatory responses; however, these processes alone do not recapitulate the complex pathology of atherosclerosis [2,4]. Therefore, the RAW 264.7 macrophage model used in this study provides a useful in vitro system for investigating the anti-inflammatory effects and molecular responses to COS in activated macrophages, but it should not be interpreted as a model demonstrating the therapeutic effects of COS on atherosclerosis.

Inflammatory activation of macrophages involves multiple interconnected signaling pathways, including NF-κB, MAPK, and nitric oxide synthase pathways [3]. In the present study, transcriptomic and functional analyses indicated that COS modulated several inflammatory processes associated with macrophage activation. To further explore potential molecular targets that may contribute to these effects, molecular docking was performed using a panel of inflammation- and atherosclerosis-associated proteins, including FGF1, HPSE, HSP90AA1, COX-2, TNF-α, inducible nitric oxide synthase (iNOS), and galectin-3. These proteins were selected based on their reported involvement in inflammatory signaling, oxidative stress, angiogenesis, extracellular-matrix remodeling, and foam-cell-associated processes [3,4,32,34]. Importantly, molecular docking was used to identify candidate protein targets and possible COS binding modes, rather than to establish direct molecular mechanisms. Experimentally characterized ligands were used as reference ligands to assess the selected binding sites. Although the predicted hydrogen-bonding and electrostatic interactions suggest potential COS interactions with inflammation-associated proteins, these results do not demonstrate physiological binding or target engagement. Therefore, the docking results should be considered hypothesis-generating evidence rather than quantitative measures of binding affinity or direct inhibition.

To move beyond predicted binding and toward expressed mechanism, transcriptomic profiling of COS-treated macrophages was cross-referenced against the docking panel. Of the receptors selected for docking, four were represented among the differentially expressed transcripts (COX-2/*Ptgs2*, inducible and endothelial nitric oxide synthase/*Nos2* and *Nos3*, and TNF-α/*Tnf*), permitting a direct comparison between predicted engagement and transcript-level response; the remaining targets were not differentially expressed in this dataset and could not be evaluated in this manner. The most coherent convergence was observed for the inducible nitric oxide synthase axis. COS suppressed nitrite accumulation in the Griess assay, and *Nos2* (iNOS), the enzymatic source of macrophage-derived NO [35], was the most strongly downregulated transcript in the dataset (log_2_FC −7.92, Q = 1.05 × 10^−26^). Together with the inclusion of NOS in the docking panel, this provides converging evidence at three levels, functional (reduced nitrite), transcriptional (reduced *Nos2*), and structure-based (predicted COS–NOS engagement), that the observed NO suppression reflects downregulation of inducible NO synthesis rather than a non-specific endpoint. A comparable convergence was found for COX-2, for which COS exhibited its highest predicted affinity, alongside concordant downregulation of *Ptgs2* (log_2_FC −0.84, FC = 0.56, Q = 5.00 × 10^−22^). The agreement between predicted catalytic-pocket occupancy and reduced transcript is consistent with a dual mode of action in which COS may both bind and limit expression of COX-2, supporting attenuation of prostaglandin-driven signaling [36]. As transcript abundance does not establish a corresponding change in protein or catalytic activity, immunoblot confirmation of iNOS and COX-2 remains necessary before structural and transcriptional concordance can be interpreted as functional inhibition.

The endothelial isoform *Nos3* (eNOS) was downregulated (log_2_FC −2.56), and because eNOS-derived NO is vasoprotective [37], its suppression is not a favorable outcome in the atherosclerotic context. This distinction is mechanistically important and indicates that the inducible and endothelial synthases must be treated as functionally separate targets rather than a single NOS entity, where the protective endothelial enzyme and the inflammatory inducible enzyme should not be conflated. In addition, although TNF-α was included in the docking panel and COS displayed moderate predicted affinity for it (−6.7 kcal/mol), the *Tnf* gene was modestly upregulated (log_2_FC +0.45) rather than suppressed, suggesting that any influence of COS on TNF-α is more plausibly exerted through direct binding or post-transcriptional mechanisms than through transcriptional repression [38]. A similar caution applies to the broader pattern of the transcriptomic data: while the terminal effectors of macrophage inflammation (iNOS, COX-2, and the IL-1β/IL-6/IL-18 cytokine axis) were coordinately downregulated in agreement with the NO result, the upstream Toll-like receptor, NF-κB, and inflammasome machinery was transcriptionally induced [39], and within the lipid-handling program, the simultaneous upregulation of the PPARγ–RXRα–CD36 axis [40] and downregulation of the ABCA1/ABCG1 efflux transporters [41], together with elevated RhoA/ROCK2 signaling [42], did not uniformly track an atheroprotective direction. The clearest favorable signals within these arms were the strong suppression of the oxidized-LDL receptor *Olr1* (LOX-1) [43] and of the chemotactic and costimulatory mediators *Ccl12*, *Ccl5*, *Cd40*, and *Mmp3* [44].

Therefore, integrating the docking predictions with transcriptomic and functional data provides stronger mechanistic support for the COX-2 and iNOS axes, where predicted interactions were accompanied by downregulation of *Ptgs2* and *Nos2* and reduced nitrite production. However, the eNOS, TNF-α, and lipid-handling results indicate that predicted interactions and transcript changes do not uniformly translate into anti-inflammatory or atheroprotective effects. Thus, the effects of COS appear to be target- and pathway-selective. Nevertheless, direct protein-level binding and target inhibition remain to be experimentally validated.

In summary, this study presented the precisely defined COS molecules obtained by HCl hydrolysis, in performing antioxidant and anti-inflammatory actions. The molecular docking of COS by strong interactions with other candidates involving macrophage activation, inflammation, and atherosclerosis-related processes, enhanced the possibility of COS to modulate inflammatory responses and pathways associated with atherosclerosis. The findings suggest that COS may modulate macrophage inflammatory responses through coordinated effects on multiple interconnected signaling pathways rather than simply suppressing inflammation through a single pathway. The strongest convergence was observed for the iNOS and COX-2 axes, supported by functional, transcriptomic, and structure-based evidence, whereas other pathways showed mixed or context-dependent responses. Thus, the effects of COS appear to be pathway- and target-selective, reflecting a potential multi-target mode of action rather than uniform suppression of inflammatory signaling. These findings provide a hypothesis-generating framework for understanding the molecular effects of COS in activated macrophages; however, direct protein-level target engagement and functional inhibition require further experimental validation. Future studies should focus on validating the effects of COS on candidate gene expression in treated macrophages and evaluating its biological activity in appropriate in vivo models to further establish its anti-inflammatory potential and underlying mechanisms.

## 4. Materials and Methods

### 4.1. Extraction and Hydrolysis of Chitosan Oligosaccharides (COS)

Shells of mud crab, *S. olivacea* were collected from Coastal Aquaculture Research and Development Regional Center 2, Samut Sakhon, Thailand. The waste crab shells were carefully rinsed and dried in an oven at 60 °C for 6 h. Subsequently, dried crab shell was homogenized in a blender into small even pieces (<20 mesh) and maintained frozen until used. The demineralization step was carried out by integrating 10% HCl 1:20 (*w*/*v*) with crab shells. The reaction proceeded at room temperature under agitation at 250 rpm for 6 h. Afterwards, the demineralized shells were filtered and washed with distilled water until neutralized. They were bleached in ethanol for 10 min and dried in an oven at 70 °C. Deproteinization step was performed by integrating 1 M NaOH with the dried demineralized shells at a solid/liquid ratio of 1:20 (*w*/*v*). The reaction was carried out under agitation at 70 °C or 75 °C for 24 h. The solid was filtered and washed with distilled water until it reached neutral pH. It was then immersed in ethanol for 10 min for further bleaching, and resulting chitin was dried in an oven at 60 °C. Chitin was deacetylated by reacting it with 12.5 M NaOH at a solid/liquid ratio of 1:20 (*w*/*v*) under agitation at 70 °C for 6 or 9 h and washed with distilled water until it neutralized. The obtained chitosan was dried in the oven at 60 °C for 4 h.

Chitosan (1 g) was then dissolved in 100 mL of 2 M HCl solution and heated at 70 °C for 2 h. The resulting solutions were then precipitated by adding 95% Ethanol in a 1:2 (*v*/*v*) ratio of COS to Ethanol, followed by incubation overnight at 4 °C to complete the precipitation. The mixture was then centrifuged at 4000× *g* for 5 min, and precipitation was collected to be washed with 70% ethanol for 5 times to remove residual acids and impurities. After that, purified products were then ultimately dried at 50 °C to obtain COS powder.

### 4.2. Characterization and Identification of COS

The molecular weight distribution of COS was determined by MALDI-TOF mass spectrometry using a SpiralTOF MALDI Imaging-TOF/TOF instrument (JMS-S3000, JEOL, Tokyo, Japan) operated in positive ion mode with 2,5-dihydroxybenzoic acid (DHB) as the matrix. Mass spectra were collected over the selected m/z ranges, and ion signals were detected mainly as protonated and sodium-associated species. The structural characteristics of COS were further confirmed by solid-state ^13^C CP/MAS NMR spectroscopy using a 400 MHz FT-NMR spectrometer (AVANCE III HD Ascend 400 WB, Bruker, Billerica, MA, USA). Spectral analysis was performed to identify characteristic carbon resonances corresponding to the glucosamine backbone and residual N-acetyl groups. Chitosan oligosaccharide lactate was used as a reference standard, described previously [14].

### 4.3. Determination of Antioxidant Property of COS

#### 4.3.1. DPPH Free Radical Scavenging Assay

The antioxidant activity of COS was determined using the 2,2-diphenyl-1-picryl hydrazyl (DPPH) radical scavenging method. A DPPH working solution (0.2 mM) was freshly prepared in ethanol according to previously reported procedures. For the assay, 100 µL of COS at different concentrations (12.5, 6.25, 3.12, 1.60, and 0.80 mg/mL) was dispensed into a 96-well microplate and mixed with 100 µL of DPPH solution. Each treatment was conducted in triplicate. Following incubation at room temperature for 30 min under protected conditions, absorbance was recorded at 517 nm using a Multiskan SkyHigh Plate Reader (Thermo Fisher Scientific, Waltham, MA, USA). Ascorbic acid (vitamin C) and Trolox were included as reference antioxidants. For the blank group, an equivalent amount of Type I water served as the substitute for the sample. Each group underwent two parallel measurements. The hydroxyl radical scavenging rate was then calculated using the following formula where A_0_ represents the absorbance of the blank control, and A_1_ represents the absorbance of the sample.

$$Hydroxyl\;radical\;scavenging\;(\%)=\frac{\;A_{1}-A_{0}}{A_{0}}\times 100$$

#### 4.3.2. ABTS Radical Scavenging Assay

ABTS radical cations were prepared by reacting 7 mM ABTS solution with 70 mM potassium persulfate at a ratio of 100:1 (*v*/*v*) and incubating the mixture in the dark at room temperature for 12–16 h. Then, the stock solution was diluted with distilled water to obtain an absorbance of approximately 0.700 at 745 nm. For the assay, 10 µL of COS at the same concentrations as described above was mixed with 100 µL of ABTS working solution in a 96-well plate and incubated in the dark for 5 min. Absorbance was then measured at 734 nm using a microplate reader. The initial control was the same as described before. The percentage of radical scavenging activity (%) was calculated using the same equation as described for the DPPH assay, and the IC_50_ value was determined from the concentration–response curve.

### 4.4. Cell Experiment

#### 4.4.1. Cell Culture

RAW 264.7 murine macrophage cells (TIB-71; ATCC, Manassas, VA, USA) were obtained from the American Type Culture Collection. Cells were cultured in complete growth medium (GM) consisting of Dulbecco’s Modified Eagle Medium with high glucose (DMEM-HG; Cytiva, South Logan, UT, USA) supplemented with 10% fetal bovine serum (FBS; ATCC, Manassas, VA, USA) and 100 units/mL penicillin plus 100 µg/mL streptomycin (P/S; Gibco, Thermo Fisher Scientific, Waltham, CA, USA). Cultures were maintained at 37 °C in a humidified atmosphere of 5% CO_2_ and 95% air. The growth medium was replaced every 2–3 days, and cells were then subcultured by gentle scraping prior to reaching confluence.

#### 4.4.2. Cytotoxicity Test Using MTT Assay

For the MTT (3-(4,5-dimethylthiazolyl-2)-2,5-diphenyltetrazolium bromide) assay, RAW 264.7 cells were seeded into 96-well plates at a density of 2 × 10^3^ cells per well in triplicate and allowed to adhere for 24 h. After incubation, the cells were treated with COS at a range of concentrations (20, 40, 80, 160, 320, 640, and 1280 µg/mL) for 24 h and 48 h. Following COS exposure, the culture medium was removed and replaced with 100 µL of 0.5 mg/mL MTT reagent (Sigma-Aldrich, Merck KGaA, Darmstadt, Germany). Cells were then incubated for 2 h at 37 °C under 5% CO_2_ to allow intracellular reduction of MTT to insoluble formazan crystals. After incubation, the MTT solution was carefully aspirated, and the formazan precipitate was dissolved with 100 µL dimethyl sulfoxide (DMSO, Merck, Merck KGaA, Darmstadt, Germany). Absorbance was measured at 570 nm with a 630 nm reference wavelength using a microplate reader (Varioskan Flash, Thermo Scientific, Waltham, MA, USA). All experiments were performed with at least five replicates. Cell viability (%) was calculated relative to vehicle control using the formula:

$$\%\;cel\;viabiity=\frac{{OD}_{COS\;treatment}-{OD}_{COS\;control\;\;\;}}{{OD}_{cell\;control}\;-\;{OD}_{medium\;control}}\;\;\times 100$$

#### 4.4.3. Determination of NO Content

Nitric oxide production in RAW 264.7 cells was quantified using the Griess reagent method (N-(1-naphthyl) ethylenediamine dihydrochloride (Component A), Sulfanilic acid (Component B) G7921). Following a 2 h treatment, cells were incubated with LPS (µg/mL) for 24 h, after which the culture supernatant was collected. For the microplate assay, 130 µL of deionized water, 20 µL of Griess reagent, and 150 µL of each sample or sodium nitrite standard were added to individual wells. The plate was incubated at room temperature for 15 min, and absorbance was measured at 548 nm. Nitric oxide levels were determined by interpolating sample absorbance values from a sodium nitrite standard curve and expressed as nitrite concentration.

### 4.5. Structural Analysis and Selection of Inflammatory Target Proteins

The SwissTargetPrediction database (http://www.swisstargetprediction.ch/; accessed 21 October 2025) was used to predict potential protein targets of COS. Targets with a predicted probability ≥0.1 were selected as a study-specific filtering criterion and further evaluated based on their relevance to inflammation-associated biological processes and the availability of suitable three-dimensional structures in the Protein Data Bank (PDB). The selected proteins included heat shock protein 90 alpha (HSP90AA1; PDB ID: 1UY6), galectin-3 (LGALS3; PDB ID: 2NN8), cyclooxygenase-2 (COX-2; PDB ID: 3LN1), tumor necrosis factor-α (TNF-α; PDB ID: 2AZ5), human inducible nitric oxide synthase (iNOS; PDB ID: 4NOS), fibroblast growth factor 1 (FGF1; also known as acidic fibroblast growth factor [aFGF]; PDB ID: 3UD8), and heparanase-1 (HPSE; an endo-β-glucuronidase; PDB ID: 5E97). COX-2, TNF-α, and iNOS are established mediators of inflammatory responses and are involved in the production or regulation of inflammatory mediators in activated macrophages [45,46]. FGF1 has been reported to suppress pro-inflammatory cytokine production and inflammation through modulation of JNK-associated signaling [47]. HPSE, an enzyme involved in heparan sulfate degradation and extracellular matrix remodeling, has also been implicated in inflammatory and other disease processes [48]. The selected protein structures were subsequently used for molecular docking analysis to evaluate the potential interactions of COS with these inflammation-associated proteins.

The 3D structures were retrieved in PDB format from the RCSB Protein Data Bank (https://www.rcsb.org/, accessed on 21 October 2025) and were virtualized using Discovery Studio (version 2024). COS ligand structure was downloaded in SDF format from PubChem (https://pubchem.ncbi.nlm.nih.gov/, accessed on 21 October 2025).

### 4.6. Molecular Docking of Chitosan-Oligosaccharide (COS) with Inflammation-Related Target Receptors

All three-dimensional structures of the target proteins and the selected ligands used and experimental ligand as positive ligand, with their SDF structures obtained from PubChem (https://pubchem.ncbi.nlm.nih.gov/, accessed on 21 October 2025). Molecular docking was performed using CB-Dock2 (https://cadd.labshare.cn/cb-dock2/index.php, accessed on 21 October 2025), which provided binding cavity parameters (A) [20] and binding docking score (kcal/mol). The resulting protein–ligand complexes were visualized and analyzed with Discovery Studio (version 2024).

### 4.7. Gene Expression of RAW 264.7 Cells by Transcriptomic Analysis

To evaluate the effect of COS on gene expression, RAW 264.7 cells were pretreated with or without COS (160 μg/mL) prior to LPS-induced inflammation, as described above. Total RNA was extracted from three independent biological replicates using TriPure™ Isolation reagent (Roche, Mannheim, Germany). Equal amounts of RNA from each replicate were pooled and submitted to BGI Genomics (Hong Kong, China) for RNA quality assessment and mRNA sequencing using the DNBseq platform (150 bp read length). Raw sequencing data were filtered to remove adapters and low-quality reads prior to alignment to the mouse reference genome using HISAT2 (version 2.0.4) and Bowtie2 (version 2.2.5). Gene expression levels were quantified as FPKM and TPM values. Differentially expressed genes (DEGs) were identified using DESeq2 (version 1.52.0) with thresholds of Q value ≤ 0.05 and log_2_FC ≥ 0.2. Functional enrichment analyses, including KEGG enrichment pathways, were performed using a hypergeometric test with significance defined at Q value ≤ 0.05 [49]. Transcriptomic analyses were conducted using the Dr. Tom platform.

## Figures and Tables

**Table 1:** Docking scores (kcal/mol) of COS with inflammation-associated receptors and their respective reference ligands.

Target/PDB ID	Cavity Size	Docking Score	Reference Ligand
FGF1 (PDB: 3UD8)	247	−4.8	heparan-sulfate-based disaccharide (NI22)
HPSE (PDB: 5E97)	1907	−6.8	2-acetamido-2-deoxy-α-D-glucopyranose-(1→4)-β-D-glucopyranuronic acid-(1→4)-2-acetamido-2-deoxy-α-D-glucopyranose-(1→4)-β-D-glucopyranuronic acid
HSP90AA1 (PDB: 1UY6)	2154	−6.0	PU3 (C_19_H_25_N_5_O_3_)
COX-2 (PDB: 3LN1)	4425	−7.7	Celecoxib
TNF-α (PDB: 2AZ5)	226	−6.4	307 (C_32_H_32_F_3_N_3_O_2_)
iNOS (PBD: 4NOS)	2099	−7.1	Ethylisothiourea (C_3_H_8_N_2_S)
Galectin-3 (PDB: 2NN8)	122	−4.8	beta-D-galactopyranose-(1-4)-beta-D-glucopyranose

**Table 2:** The differential expression of genes related to inflammatory pathways.

Expression	Gene	Gene Description	Pathway	Accession Number	Log_2_FC	*p* Value	Q Value
Up regulation	*Nlrp3*	NLR family pyrin domain containing 3	Lipid and atherosclerosis; NOD-like receptor signaling	NM_145827	+9.67	4.38 × 10^−72^	3.32 × 10^−71^
	*Tlr6*	toll-like receptor 6	Lipid and atherosclerosis; Toll-like receptor signaling	NM_001359180	+9.65	2.33 × 10^−71^	1.75 × 10^−70^
	*Tab2*	TGF-beta activated kinase 1/MAP3K7 binding protein 2	Lipid and atherosclerosis; MAPK signaling; TNF signaling; NF-κB signaling; NOD-like receptor signaling; Toll-like receptor signaling	NM_001359534	+6.73	6.57 × 10^−14^	1.62 × 10^−13^
	*Ikbkg*	inhibitor of kappa B kinase gamma	Lipid and atherosclerosis; MAPK signaling; TNF signaling; NF-κB signaling; NOD-like receptor signaling; Toll-like receptor signaling	NM_001161422	+6.44	9.01 × 10^−12^	2.04 × 10^−11^
	*Traf3*	TNF receptor-associated factor 3	Lipid and atherosclerosis; NF-κB signaling; TNF signaling; NOD-like receptor signaling; Toll-like receptor signaling	NM_001286122	+5.76	5.66 × 10^−8^	1.09 × 10^−7^
	*Cxcl3*	chemokine (C-X-C motif) ligand 3	Lipid and atherosclerosis; NF-κB signaling; TNF signaling; NOD-like receptor signaling	NM_203320	+4.26	1.03 × 10^−5^	<10^−300^
	*Jak2*	Janus kinase 2	Lipid and atherosclerosis	NM_001048177	+3.34	1.18 × 10^−99^	1.18 × 10^−98^
	*Mapk8*	mitogen-activated protein kinase 8	Lipid and atherosclerosis; MAPK signaling; TNF signaling; NOD-like receptor signaling; Toll-like receptor signaling	NM_016700	+3.17	5.78 × 10^−13^	1.37 × 10^−12^
	*Tlr4*	toll-like receptor 4	HIF-1 signaling; NF-κB signaling; Lipid and atherosclerosis; NOD-like receptor signaling; Toll-like receptor signaling	NM_021297	+3.12	3.38 × 10^−268^	1.00 × 10^−266^
	*Map2k4*	mitogen-activated protein kinase kinase 4	Lipid and atherosclerosis; MAPK signaling; TNF signaling; Toll-like receptor signaling	NM_001316367	+2.89	9.05 × 10^−19^	2.60 × 10^−18^
	*Map3k7*	mitogen-activated protein kinase kinase kinase 7	Lipid and atherosclerosis; MAPK signaling; TNF signaling; NF-κB signaling; NOD-like receptor signaling; Toll-like receptor signaling	NM_009316	+2.32	3.12 × 10^−102^	3.24 × 10^−101^
	*Ikbkb*	inhibitor of kappa B kinase beta	Lipid and atherosclerosis; MAPK signaling; TNF signaling; NF-κB signaling; NOD-like receptor signaling; Toll-like receptor signaling	NM_001159774	+1.77	1.74 × 10^−80^	1.44 × 10^−79^
	*Traf6*	TNF receptor-associated factor 6	Lipid and atherosclerosis; MAPK signaling; NF-κB signaling; NOD-like receptor signaling; Toll-like receptor signaling	NM_001303273	+1.62	3.26 × 10^−43^	1.61 × 10^−42^
	*Nfe2l2*	nuclear factor, erythroid derived 2, like 2	Lipid and atherosclerosis	NM_010902	+1.59	9.66 × 10^−203^	2.10 × 10^−201^
	*Tlr2*	toll-like receptor 2	Lipid and atherosclerosis; Toll-like receptor signaling	NM_011905	+1.46	2.06 × 10^−134^	2.88 × 10^−133^
	*Map3k5*	mitogen-activated protein kinase kinase kinase 5	Lipid and atherosclerosis; MAPK signaling; TNF signaling	NM_008580	+1.40	1.88 × 10^−23^	6.10 × 10^−23^
	*Fos*	FBJ osteosarcoma oncogene	Lipid and atherosclerosis; MAPK signaling; TNF signaling; Toll-like receptor signaling	NM_010234	+1.36	3.04 × 10^−51^	1.70 × 10^−50^
	*Irak4*	interleukin-1 receptor-associated kinase 4	Lipid and atherosclerosis; MAPK signaling; NF-κB signaling; NOD-like receptor signaling; Toll-like receptor signaling	NM_029926	+1.30	6.04 × 10^−26^	2.08 × 10^−25^
	*Ccl2*	chemokine (C-C motif) ligand 2	Lipid and atherosclerosis; NOD-like receptor signaling; TNF signaling	NM_011333	+1.19	7.08 × 10^−4^	<10^−300^
	*Casp1*	caspase 1	Lipid and atherosclerosis; NOD-like receptor signaling	NM_009807	+0.92	1.52 × 10^−85^	1.33 × 10^−84^
	*Ncf2*	neutrophil cytosolic factor 2	Lipid and atherosclerosis	NM_010877	+0.79	8.50 × 10^−184^	1.66 × 10^−182^
	*Tnf*	tumor necrosis factor	Lipid and atherosclerosis; MAPK signaling; NF-κB signaling; NOD-like receptor signaling	NM_013693	+0.45	2.35 × 10^−127^	3.08 × 10^−126^
	*Rela*	v-rel reticuloendotheliosis viral oncogene homolog A (avian)	HIF-1 signaling; MAPK signaling; Lipid and atherosclerosis; NF-κB signaling; TNF signaling; NOD-like receptor signaling; Toll-like receptor signaling	NM_009045	+0.42	6.62 × 10^−11^	1.45 × 10^−10^
	*Cybb*	cytochrome b-245, beta polypeptide	HIF-1 signaling; Lipid and atherosclerosis; NOD-like receptor signaling	NM_007807	+0.32	3.12 × 10^−88^	2.80 × 10^−87^
**Down** **regulation**	*Nos2*	nitric oxide synthase 2, inducible	HIF-1 signaling	NM_001313922	−7.92	2.96 × 10^−27^	1.05 × 10^−26^
	*Mapk14*	mitogen-activated protein kinase 14	Lipid and atherosclerosis; MAPK signaling; TNF signaling; NOD-like receptor signaling; Toll-like receptor signaling	NM_001168508	−5.74	8.71 × 10^−12^	1.97 × 10^−11^
	*Ccl5*	chemokine (C-C motif) ligand 5	Lipid and atherosclerosis; NOD-like receptor signaling; TNF signaling; Toll-like receptor signaling	NM_013653	−5.42	4.02 × 10^−4^	<10^−300^
	*Il1b*	interleukin 1 beta	Lipid and atherosclerosis; MAPK signaling; TNF signaling; NF-κB signaling; NOD-like receptor signaling; Toll-like receptor signaling	NM_008361	−2.94	2.22 × 10^−4^	<10^−300^
	*Nos3*	nitric oxide synthase 3, endothelial cell	HIF-1 signaling; Lipid and atherosclerosis	NM_008713	−2.56	2.35 × 10^−7^	4.36 × 10^−7^
	*Nfkbia*	nuclear factor of kappa light polypeptide gene enhancer in B cells inhibitor, alpha	Lipid and atherosclerosis; NF-κB signaling; TNF signaling NOD-like receptor signaling; Toll-like receptor signaling	NM_010907	−2.14	5.11 × 10^−5^	<10^−300^
	*Il6*	interleukin 6	HIF-1 signaling; TNF signaling; Lipid and atherosclerosis; NOD-like receptor signaling; Toll-like receptor signaling	NM_001314054	−1.80	2.16 × 10^−7^	4.03 × 10^−7^
	*Il18*	interleukin 18	Lipid and atherosclerosis; NOD-like receptor signaling	NM_001357221	−1.94	3.30 × 10^−45^	1.68 × 10^−44^
	*Mapk3*	mitogen-activated protein kinase 3	HIF-1 signaling; MAPK signaling; Lipid and atherosclerosis; NOD-like receptor signaling; TNF signaling; Toll-like receptor signaling	NM_011952	−1.59	3.24 × 10^−6^	<10^−300^
	*Cyba*	cytochrome b-245, alpha polypeptide	Lipid and atherosclerosis; NOD-like receptor signaling	NM_007806	−1.44	1.37 × 10^−25^	4.67 × 10^−25^
	*Tnfrsf1a*	tumor necrosis factor receptor superfamily, member 1a	Lipid and atherosclerosis; MAPK signaling; NF-κB signaling; TNF signaling	NM_011609	−1.05	4.43 × 10^−97^	4.34 × 10^−96^
	*Ptgs2*	prostaglandin-endoperoxide synthase 2	NF-κB signaling	NM_011198	−0.84	3.63 × 10^−7^	5.00 × 10^−22^

**Table 3:** The differential expression of genes related to foam cell formation-related pathways.

Expression	Gene	Gene Description	Pathway	Accession Number	Log_2_FC	*p* Value	Q Value
**Up** **regulation**	*Rxra*	retinoid X receptor alpha	Lipid and atherosclerosis	NM_001290481	+9.29	1.55 × 10^−58^	9.67 × 10^−58^
	*Nfatc2*	nuclear factor of activated T cells, cytoplasmic, calcineurin dependent 2	Lipid and atherosclerosis	NM_001291171	+7.94	1.09 × 10^−27^	3.92 × 10^−27^
	*Nfatc1*	nuclear factor of activated T cells, cytoplasmic, calcineurin dependent 1	Lipid and atherosclerosis; MAPK signaling	NM_198429	+6.00	5.58 × 10^−11^	1.23 × 10^−10^
	*Pparg*	peroxisome proliferator activated receptor gamma	Lipid and atherosclerosis	NM_001308352	+5.24	6.63 × 10^−6^	1.13 × 10^−5^
	*Ppp3cb*	protein phosphatase 3, catalytic subunit, beta isoform	Lipid and atherosclerosis; MAPK signaling	NM_008914	+3.97	1.11 × 10^−80^	9.19 × 10^−80^
	*Nfatc3*	nuclear factor of activated T cells, cytoplasmic, calcineurin dependent 3	Lipid and atherosclerosis; MAPK signaling	NM_001368797	+2.47	5.81 × 10^−25^	1.95 × 10^−24^
	*Cd36*	CD36 molecule	Lipid and atherosclerosis	NM_007643	+2.21	6.73 × 10^−4^	<10^−300^
**Down** **regulation**	*Abca1*	ATP-binding cassette, sub-family A (ABC1), member 1	Lipid and atherosclerosis	NM_013454	−0.89	9.52 × 10^−6^	1.60 × 10^−5^
	*Abcg1*	ATP binding cassette subfamily G member 1	Lipid and atherosclerosis	NM_009593	−1.11	2.09 × 10^−27^	7.45 × 10^−27^
	*Ldlr*	low density lipoprotein receptor	Lipid and atherosclerosis	NM_001252659	−1.41	7.16 × 10^−27^	2.52 × 10^−26^
	*Olr1*	oxidized low density lipoprotein (lectin-like) receptor 1	Lipid and atherosclerosis	NM_138648	−4.92	6.61 × 10^−5^	1.05 × 10^−4^

**Table 4:** The differential expression of genes related to lipid- and atherosclerosis-associated pathways.

Expression	Gene	Gene Description	Pathway	Accession Number	Log_2_FC	*p* Value	Q Value
**Up** **regulation**	*Mib2*	mindbomb E3 ubiquitin protein ligase 2	Lipid and atherosclerosis	NM_001369166	+7.68	5.99 × 10^−24^	1.97 × 10^−23^
	*Pou2f1*	POU domain, class 2, transcription factor 1	Lipid and atherosclerosis	NM_198933	+6.89	2.74 × 10^−15^	7.08 × 10^−15^
	*Rhoa*	ras homolog family member A	Lipid and atherosclerosis; NOD-like receptor signaling	NM_001313961	+3.42	6.27 × 10^−29^	2.31 × 10^−28^
	*Pik3ca*	phosphatidylinositol-4,5-bisphosphate 3-kinase catalytic subunit alpha	HIF-1 signaling; Lipid and atherosclerosis; TNF signaling; Toll-like receptor signaling	NM_008839	+2.66	1.19 × 10^−239^	3.09 × 10^−238^
	*Vav2*	vav guanine nucleotide exchange factor 2	Lipid and atherosclerosis	NM_009500	+2.33	1.48 × 10^−35^	6.29 × 10^−35^
	*Vav3*	vav guanine nucleotide exchange factor 3	Lipid and atherosclerosis	NM_020505	+2.31	6.25 × 10^−92^	5.82 × 10^−91^
	*Rock2*	Rho-associated coiled-coil containing protein kinase 2	Lipid and atherosclerosis	NM_009072	+2.24	9.64 × 10^−183^	1.88 × 10^−181^
	*Cdc42*	cell division cycle 42	Lipid and atherosclerosis; MAPK signaling	NM_001243769	+1.81	1.66 × 10^−22^	5.27 × 10^−22^
	*Src*	Rous sarcoma oncogene	Lipid and atherosclerosis; MAPK signaling—fly	NM_009271	+1.02	1.74 × 10^−3^	2.48 × 10^−3^
**Down** **regulation**	*Akt2*	thymoma viral proto-oncogene 2	HIF-1 signaling; TNF signaling; MAPK signaling; Lipid and atherosclerosis; Toll-like receptor signaling	NM_001331109	−9.73	8.39 × 10^−74^	6.46 × 10^−73^
	*Pik3cd*	phosphatidylinositol-4,5-bisphosphate 3-kinase catalytic subunit delta	HIF-1 signaling; Lipid and atherosclerosis; TNF signaling; Toll-like receptor signaling	NM_001029837	−7.33	1.78 × 10^−19^	5.22 × 10^−19^
	*Ptk2*	PTK2 protein tyrosine kinase 2	Lipid and atherosclerosis	NM_001358046	−6.42	1.37 × 10^−11^	3.09 × 10^−11^
	*Irf7*	interferon regulatory factor 7	Lipid and atherosclerosis; NOD-like receptor signaling; Toll-like receptor signaling	NM_016850	−4.07	2.53 × 10^−5^	<10^−300^
	*Mmp3*	matrix metallopeptidase 3	Lipid and atherosclerosis; TNF signaling	NM_010809	−4.33	1.49 × 10^−3^	2.13 × 10^−3^
	*Ccl12*	chemokine (C-C motif) ligand 12	Lipid and atherosclerosis; NOD-like receptor signaling; TNF signaling	NM_011331	−3.77	2.02 × 10^−37^	8.91 × 10^−37^
	*Cd40*	CD40 antigen	Lipid and atherosclerosis; NF-κB signaling; Toll-like receptor signaling	NM_170703	−3.59	1.55 × 10^−74^	1.20 × 10^−73^
	*Arhgef1*	Rho guanine nucleotide exchange factor (GEF) 1	Lipid and atherosclerosis	NM_001130150	−2.58	9.89 × 10^−32^	3.87 × 10^−31^

## Data Availability

The original contributions presented in this study are included in this article. Further inquiries can be directed to the corresponding author.

## Supplementary Materials

The following supporting information can be downloaded at: https://www.mdpi.com/article/10.3390/ijms27177775/s1.
