# Supplementary material for: Chitosan Oligosaccharides Modulate Macrophage Inflammatory Signaling: Molecular Docking and Transcriptomic Evidence
Source: Int J Mol Sci. 2026 Aug 30;27(17):7775. doi: 10.3390/ijms27177775 (PMC13566840; doi:10.3390/ijms27177775)
Supplement: Supplementary file 1 [file ijms-27-07775-s001.zip › Supplementary figures.pdf]

Chitosan Oligosaccharides Modulate Macrophage Inflammatory Signaling: Molecular Docking and Transcriptomic Evidence

Yujun Sung [1], Siriporn Namwongsa [1,2], Sineenart Songkoomkrong [1,3], Supawadee Duangprom [1,3] and Napamanee Kornthong [1,3,*]

1 Research Unit in Innovative Marine Biotechnology and Natural Bio-Resources for Sustainable Health and Wellness, Thammasat University, Pathum Thani 12120, Thailand; ethansung416@gmail.com (Y.S.); siriphorn.nonkhaow@gmail.com (S.N.); sineenartsong@gmail.com (S.S.); su.duangprom@gmail.com (S.D.)
2 Department of Biochemistry and Genetic Engineering, Faculty of Medicine, Kasetsart University, Bangkok 10900, Thailand
3 Chulabhorn International College of Medicine, Thammasat University, Rangsit Campus, Pathum Thani 12120, Thailand
* Correspondence: napamaneenatt@gmail.com or napanatt@tu.ac.th

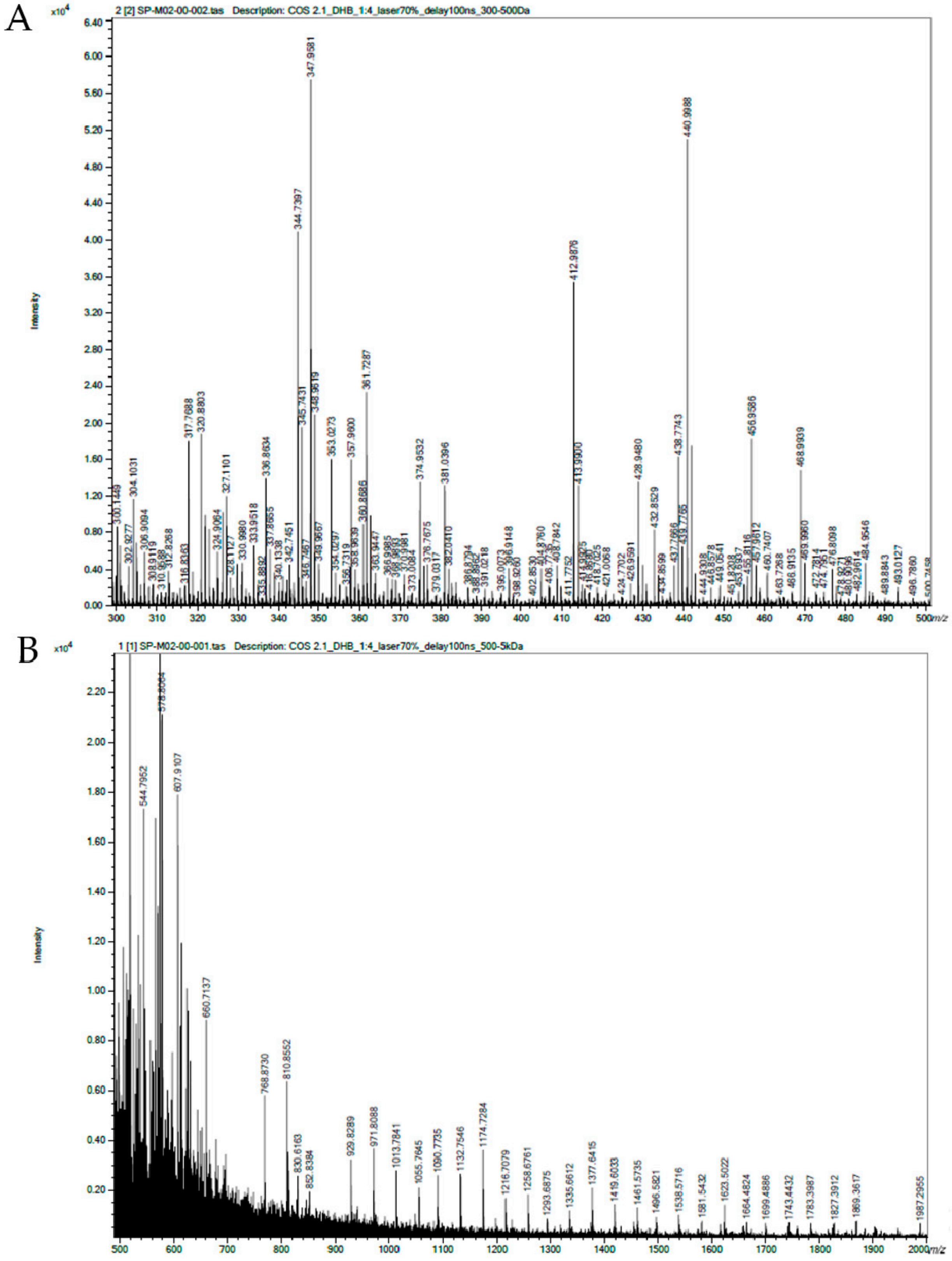

Figure S1. (A) Positive ion MALDI-TOF MS mass spectrum of the mixture of COS (with DHB matrix) in the range 300 to 500 Da and **(B)** 500 to 5000 Da.

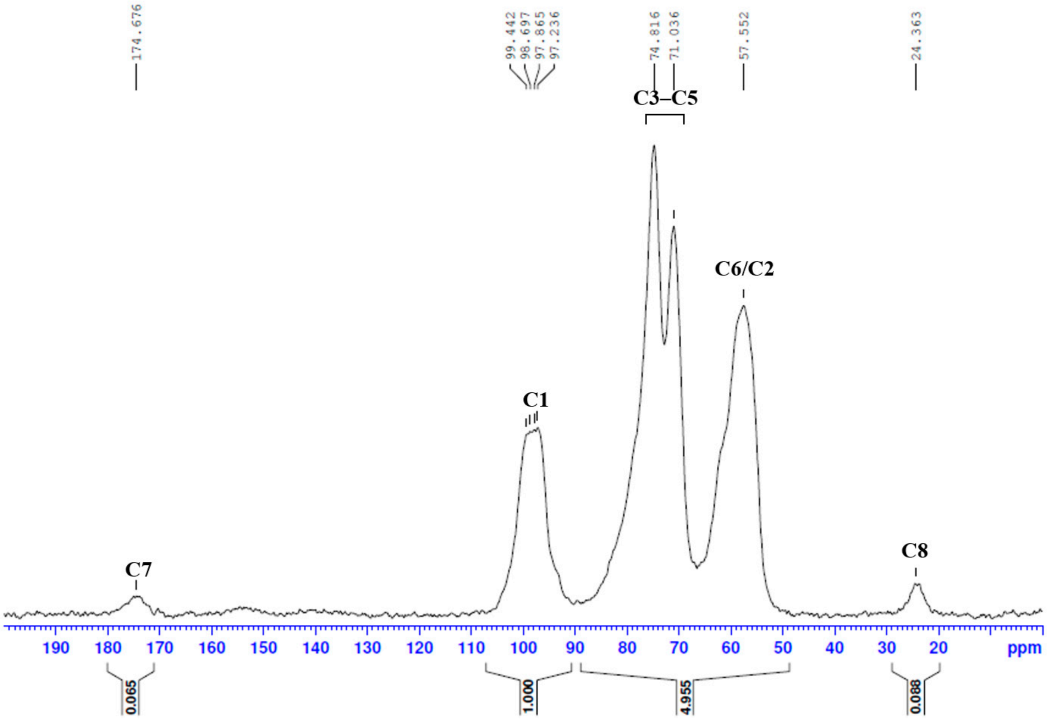

Figure S2. ^{13}C NMR spectrum (400 MHz) of COS.
